# Interleukin-17 aggravates right ventricular remodeling via activating STAT3 under both normoxia and hypoxia

**DOI:** 10.1186/s12872-021-02069-4

**Published:** 2021-05-21

**Authors:** Jing Huang, Wei Zhang, Cai-lian Zhang, Lei Wang

**Affiliations:** 1 Department of Rheumatism and Immunology, First Affiliated Hospital of Xi’an Jiaotong University, Xi’an, 710061 People’s Republic of China; 2 Department of Emergency Medicine, First Affiliated Hospital of Xi’an Jiaotong University, Xi’an, 710061 People’s Republic of China; 3 Department of Pulmonary and Critical Care Medicine, Yanan University Affiliated Hospital, Yanan, 716000 People’s Republic of China; 4 Department of Pulmonary and Critical Care Medicine, Second Affiliated Hospital of Xi’an Jiaotong University, No. 157 Xiwu Road, Xin Cheng District, Xi’an, 710004 People’s Republic of China

**Keywords:** Right ventricular hypertrophy, cardiomyocyte apoptosis, IL-17, STAT3

## Abstract

**Objective:**

Proinflammatory cytokine interleukin 17 (IL-17) is involved in ventricular remodeling, mainly of the left ventricle. This study was designed to explore the role of IL-17 played in the pathogenesis of right ventricular hypertrophy (RVH), aiming to provide a novel treatment target or diagnostic biomarker options for improving the care of RVH patients.

**Methods:**

C57BL/6 mice were maintained in 10% O_2_ chamber or room air for four weeks. Right ventricular hypertrophy index (RVHI), RV/body weight ratio, pulmonary arteriolar remodeling determined by percent media thickness (%MT), and the cardiomyocyte diameter of RV were evaluated. Mice were treated with exogenous recombinant mouse IL-17 (rmIL-17, 1 μg per dose twice a week) for four weeks. H9c2 cardiomyocytes were cultured and treated with IL-17 (10 ng/mL) and STAT3 inhibitor (10 ng/mL) either under normoxia (21% O_2_, 5% CO_2_, 74% N_2_) or under hypoxia (3% O_2_, 5% CO_2_, 92% N_2_). Cardiomyocyte viability was assessed by Cell counting kit 8 (CCK-8) assay. The mRNA level was detected by RT-PCR, where as the protein expression was measured by Western blot, immunohistochemistry, and immunofluorescent analyses.

**Results:**

In vivo experiments showed that IL-17 did not affect the pulmonary artery under normoxia, after treatment with rmIL-17, %MT was not changed, while RVHI and the RV/body weight ratio were increased, indicating that IL-17 directly induced right ventricular hypertrophy. In a time-course study, the mice were exposed to hypoxia for 0, 1, 2, 3, 4 weeks, respectively. We found that the expression of IL-17 was gradually upregulated in RV tissue in a time-dependent manner after one week of hypoxia exposure, especially at the third and fourth week. Cardiomyocyte hypertrophy and apoptosis were observed after the exposure of the mice to hypoxia for four weeks, rmIL-17 further aggravated the hypoxia-induced cardiomyocyte hypertrophy and apoptosis. The expression of p-STAT3 in the IL-17-deficient mice was lower than in the wild-type mice. In vitro, IL-17 inhibited cardiomyocyte viability and induced cardiomyocyte apoptosis via STAT3 under both normoxic and hypoxic conditions.

**Conclusions:**

These findings support a role for IL-17 as a mediator in the pathogenesis RVH, which might be considered as a potential novel anti-inflammation therapeutic strategy or diagnostic biomarker for RVH.

**Supplementary Information:**

The online version contains supplementary material available at 10.1186/s12872-021-02069-4.

## Introduction

Right ventricular hypertrophy (RVH) results from adaptation of right ventricle (RV) to an increased afterload and biomechanical stress, ultimately leading to right heart failure (RHF) and death, mainly observed in pulmonary hypertension [1]. The main pathophysiological characteristics of RVH are cardiomyocyte hypertrophy, elevated capillary density and synthesis of extracellular matrix proteins [2], which is a remodeling process. In contrast to the left ventricle (LV), RV rapidly switches from adaptive to maladaptive RVH and finally to end-stage heart failure, leading eventually to death. However, no specific biomarkers for RV failure and no treatment have been indentified so far that specifically address RV dysfunction. Therefore, an urgent need exists to clarify the potential pathogenesis of RVH.

RVH is a complex and heterogeneous disease, and the RV remodeling of this disease is not well elucidated. Many factors are reportedly involved in RVH, including ion channels, neurohormonal activation, metabolism dysfunction, myocardial perfusion, genetic factors, inflammation and extracellular matrix changes [2]. Among them, inflammation and immune activation critically involved in pulmonary vascular and RV remodeling [3, 4]. Furthermore, inflammation, immune mediator and immune cell activities, such as neutrophils, macrophage and lymphocytes infiltration, contribute to the remodeling and the development of RVH [5–8].

Evidence suggested that CD4^+^ helper T (Th) cells participate in inflammatory heart disease, and the deficiency in CD4^+^ Th cells induced ventricular remodeling [9, 10]. The number of CD4^+^ Th cells subset Th17 cells infiltrated in the LV was increased significantly in the heart failure rabbit model [9]. The important pro-inflammation cytokine IL-17 mainly secreted by Th17 cells, has been reported to participate in the cardiac ventricular remodeling in some heart diseases, such as ischemic heart failure [9], myocardial infarction [11], and dilated cardiomyopathy [12]. While previous studies have predominantly focused on the role of IL-17 in the left ventricle remodeling, very few studies have investigated its involvement in RVH. Thus, the role of IL-17 in RVH remains to be elucidated (Additional file 1).

Therefore, in this study, we examined the role of IL-17 in the pathogenesis of RV remodeling and the possible signal pathways involved to provide a novel treatment target or diagnostic biomarker options for improving the care of RVH patients.

## Materials and methods

### Animal models

The experimental animals were approved by the Institutional Animal Care and Use Committee of Xi’an jiaotong University. Animals were housed in accordance with the regulations for the Management of Laboratory Animals published by the Ministry of Science and Technology of the People’s Republic of China. Eight-week old male C57BL/6N mice weighing 20–25 g were bought from Vital River Laboratory Animal Technology Company (Beijing, China), *IL17*^−/−^ male mice (C57BL/6N background) aged 8 weeks and their matched wide type (WT) littermates were a kindly gift from Professor Dai of China-Japan Friendship Hospital (Beijing, China). All the mice were housed in a 12 h light/12 h dark cycle, and food and water were available ad libitum. Mice were allowed to acclimate for 3 days and were then exposed to normobaric hypoxia (10% O_2_) in an airtight plexiglass chamber for 4 weeks. Control mice were exposed to room air under the same conditions.

### Treatments

For exogenous IL-17 administration experiments, recombinant murine IL-17 (rmIL-17, peprotech, NJ, USA) dissolved in PBS was administered intraperitoneally at a dose of 1 μg/mouse per dose twice a week for consecutive 4 weeks, the control groups were given equal volume of PBS [13]. For experiments of different time points for hypoxia, the mice were sacrificed at 0, 1, 2, 3, or 4 weeks, respectively. The flowcharts of in vivo experiments can be seen in Additional file 1.

### Cells culture

H9c2 cardiomyocytes were purchased from Procell Life Science & Technology (CL-0089, Wuhan, China) and subsequently kept in DMEM with 10% (v/v) FBS, 100 U/mL penicillin and 100 g/mL streptomycin in a humidified normoxia condition (21% O_2_, 5% CO_2_, 74% N_2_) at 37 °C. Cells were passaged (passages 4–6) after reaching 80–90% confluence, detached with 0.05% trypsin, 0.04% EDTA (Sigma-Aldrich, MO, USA) in phosphate-buffered saline (PBS). For hypoxia injury, the cells were maintained at 37 °C in a humidified hypoxia condition (3% O_2_, 5%CO_2_, 92% N_2_) for 24 h, and treated with IL-17 (10 ng/ml) or STAT3 inhibitor (10 ng/ml).

### CCK-8 assay

To evaluate cell viability, H9c2 cells were seeded in 96-well plates and tested via CCK-8 assay (Dojindo, Kumamoto, Japan). After addition 10 ul of CCK-8 solution in each well, cells were then cultured. After 4 h, the absorbance of 450 nm was monitored via microplate reader (Thermo, MULTISKAN MK3).

### Measurement of right ventricular hypertrophy

The method was described in previous papers [14, 15]. Mice were anesthetized with 2% pentobarbital (50 mg/kg, i.p.). The mice were then sacrificed, and the hearts were collected. To evaluate the extent of right ventricle (RV) hypertrophy, RV tissues were separated and the weights of the RV and the left ventricle (LV) plus interventricular septum (S) were measured respectively. The right ventricular hypertrophy index (RVHI) was defined as: RVHI (%) = [RV/(LV + S)] × 100. The ratio of RV weight to body weight (BW) was also calculated.

Cardiomyocyte cross-sectional diameter was determined in the RV as measures of cardiac ventricle tissue remodeling. 4 μm sections of the RV were stained with H&E. Microscopic images were analyzed in a blinded manner using Nikon microscope digital camera system and its image analysis program (Nikon, Tokyo, Japan).

### Lung artery morphometric analysis

The method was described in previous papers [13, 16]. The paraffin-embedded lungs were serially sectioned at a thickness of 4 um for morphometric analysis. Images of pulmonary arterioles were captured with an Olympus microscope digital camera system (Olympus, Tokyo, Japan), and the arterial circumferences were measured using the Image Pro Plus 5.1 image analysis program (Media Cybernetics, Silver Spring, MD). The pulmonary arterioles with external diameters smaller than 100 μm accompanied by either alveolar ducts or alveoli were measured. Pulmonary arteriolar remodeling was estimated by percent media thickness (MT%), MT% = (circumference_ext_/*π*-circumferenceint_int_/*π*)/(circumference_ext_/*π*) × 100 [17], and circumference_ext_ and circumference_int_ mean the circumferences bounded by the external and internal elastic lamina.

### RT-PCR

RT-PCR was performed as previously conducted in our laboratory [16]. Total RNA was extracted from cultured cells using Trizol reagents (Sigma-Aldrich, USA). Reverse transcription was performed using Superscript III First-strand Synthesis System (Invitrogen, USA) and quantitative, real-time PCR with Power SYBR Green PCR Master Mix (Applied Biosystems, UK). The relative abundance of target mRNA was normalized to that of the GAPDH, by a comparative cycle threshold method (2^−ΔΔCT^). The primer sequences were as listed below:

GAPDH: Forward: 5′-ACAGCAACAGGGTGGTGGAC -3′, and Reverse: 5′-TTTGAGGGTGCAGCGAACTT-3′;

Bax: Forward: 5′-CAGGCGAATTGGCGATGAAC -3′, and Reverse: 5′-CCCAGTTGAAGTTGCCGTCT-3′;

Bcl2: Forward: 5′-GCCTTCTTTGAGTTCGGTGG -3′, and Reverse: 5′-CTGAGCAGCGTCTTCAGAGA -3′;

Caspase-3: Forward: 5′-TGGACTGCGGTATTGAGACA -3′, and Reverse: 5′-GCGCAAAGTGACTGGATGAA-3′.

### Immunohistochemical analysis

IL-17 expression levels were detected in paraffin-embedded mouse right ventricular tissue sections using the rabbit anti-mouse IL-17 (Abcam, UK, 1:50 dilution) antibodies. Protein expression was visualized using HRP-conjugated goat anti-rabbit secondary antibody (R&D, CA, USA, 1:200 dilution). The positive cells were developed by diaminobenzidine (DAB) reagent, nuclei were counterstained with hematoxylin.

### Immunofluorescent analysis

IL-17, Bax and caspase-3 expression levels were detected in paraffin-embedded RV sections using the anti-IL17 antibody (Abcam, UK, 1:20 dilution), anti-Bax antibody (Proteintech, Wuhan, China, 1:100 dilution) and anti-caspase-3 antibody (Bioss, Beijing, China, 1:200 dilution), protein expression was visualized using Alex Fluor 488 Goat Anti-Rabbit secondary antibodies (R&D, CA, USA, 1:200 dilution, green) or Cy3 Conjugated Goat Anti-Rabbit IgG secondary antibody (BOSTER biological technology, Wuhan, China, 1:100 dilution, red). Nuclei were counterstained with DAPI.

### Western blot analysis

Proteins were separated by SDS-PAGE and transferred to nitrocellulose membranes. Membranes were blocked in 5% non-fat dry milk for 1 h, followed by incubation in anti-IL-17 (rabbit, Affinity Biosciences, OH, USA, 1:1000 dilution), anti-p STAT3 (rabbit, Affinity Biosciences, OH, USA, 1:1000 dilution), anti-GAPDH (rabbit, Goodhere Biological Technology, China, 1:1000 dilution), anti-Bax (rabbit, proteintech, Wuhan, China, 1:3000 dilution), anti-Bcl2 (rabbit, proteintech, Wuhan, China, 1:600 dilution) and anti-caspase3 (rabbit, Cell Signaling Technology, MA, USA, 1:1000 dilution) antibodies overnight at 4 °C. After the overnight incubation, membranes were washed with TBST buffer and re-incubated with a goat anti-rabbit IgG-HRP secondary antibody. The membranes were washed with TBST buffer and reacted with the electrochemiluminescence (ECL) substrate (Thermo, MA, USA) and exposed to X-ray film. The value of the relative density of each target protein band was normalized to the density of the corresponding GAPDH band.

### Statistical analysis

Data were presented as means ± SD. Unpaired Student’s t-test was used for comparisons between two groups. One-way ANOVA with the Bonferroni’s multiple comparisons test was used to evaluate differences between more than two groups. *p* < 0.05 was considered statistically significant.

## Results

### Under normoxia, IL-17 induced right ventricular hypertrophy but not pulmonary vascular remodeling

**Fig. 1:**
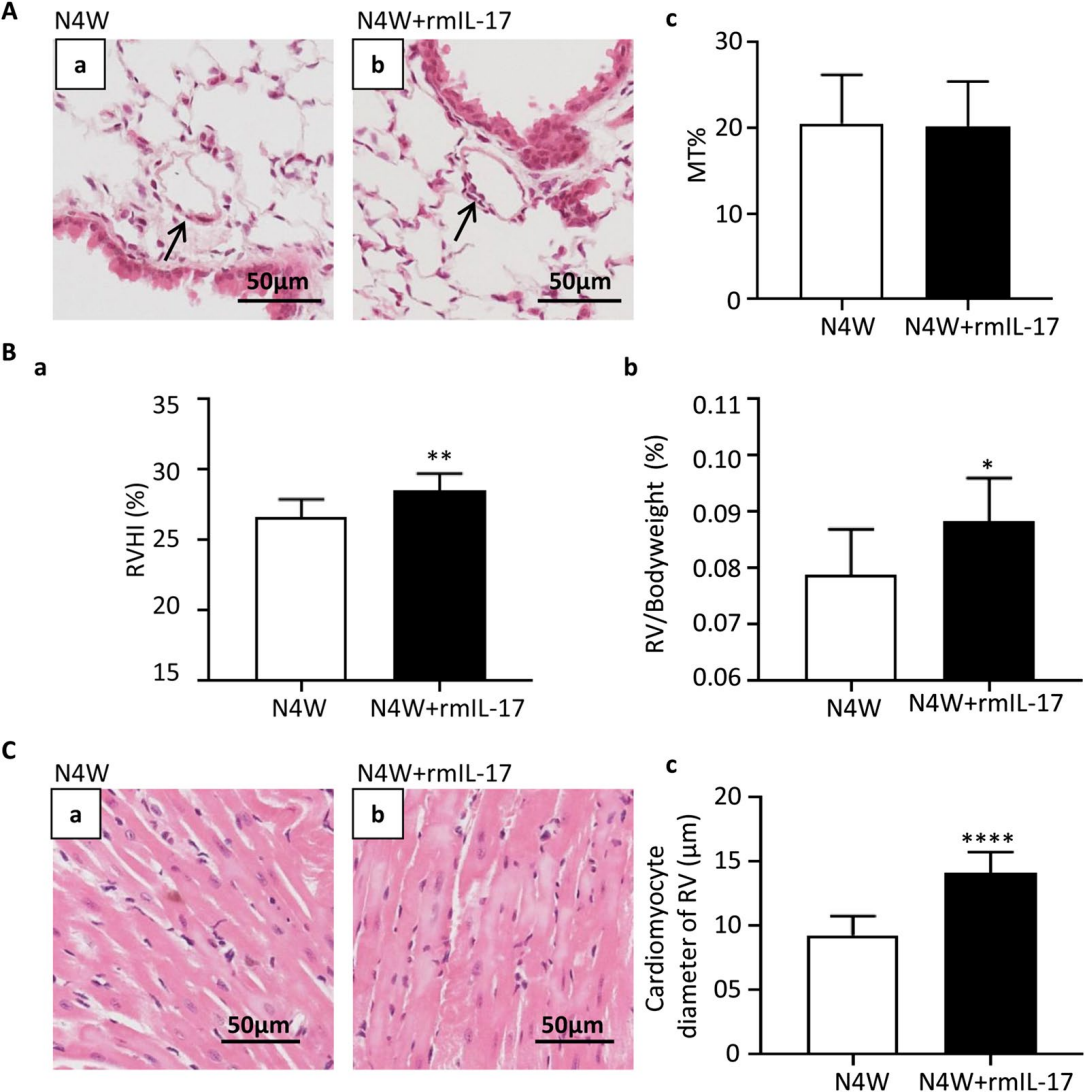
IL-17 induces right ventricular hypertrophy under normoxia. **A** Comparison of pulmonary vascular remodeling in mice under conditions of normoxia for 4 weeks (N4W, n = 9) with or without recombinant mouse IL-17 (rmIL-17) (N4W + rmIL-17, n = 9). (a, b) H&E staining of pulmonary arterioles. The arrows show pulmonary arterioles. Scale bars: 50 μm. (c) Percentage medial thicknesses (MT%) of pulmonary arterioles less than 100 μm. **B** Right ventricular hypertrophy of mice in the groups of N4W and N4W + rmIL-17. (a) RVHI of mice. RVHI: right ventricular hypertrophy index. **p = 0.0053. (b) RV/body weight ratio of mice. *p = 0.0220. **C** Cardiomyocyte hypertrophy of the right ventricular tissue sections derived from mice in the groups of N4W and N4W + rmIL-17. (a, b) Representative H&E staining. (c) Bar chart for cardiomyocytes diameter in the right ventricle of each group. ****p < 0.0001

We previously confirmed that IL-17 aggravated hypoxia-induced pulmonary vascular remodeling and the secondary right ventricular hypertrophy (RVHI and RV/body weight ratio) that resulted from increased afterload [13]. Here, we investigated whether IL-17 induced pulmonary vascular remodeling under normoxia and its effect on right ventricular. After treating the mice with recombinant mouse IL-17 (rmIL-17) for four weeks, pulmonary artery morphometrics data were analyzed. Vascular remodeling of PH was observed mainly in the distal pulmonary arteriole, and thus % MT of < 100 μm in the intra-pulmonary arterioles were calculated. The results (Fig. 1A) showed that rmIL-17 did not affect the % MT of the pulmonary arterioles under normoxia. We also evaluated the right ventricle of the mice. Interestingly, RVHI and RV/body weight ratio, two of the indexes of the right ventricular hypertrophy, were significantly increased after the mice under normoxia treated with rmIL-17, as showed in the Fig. 1B. Compared with normoxia mice, rmIL-17 treatment increased the diameter of cardiomyocyte significantly (Fig. 1C).

### The expression of IL-17 was upregulated during the chronic hypoxia exposure

**Fig. 2:**
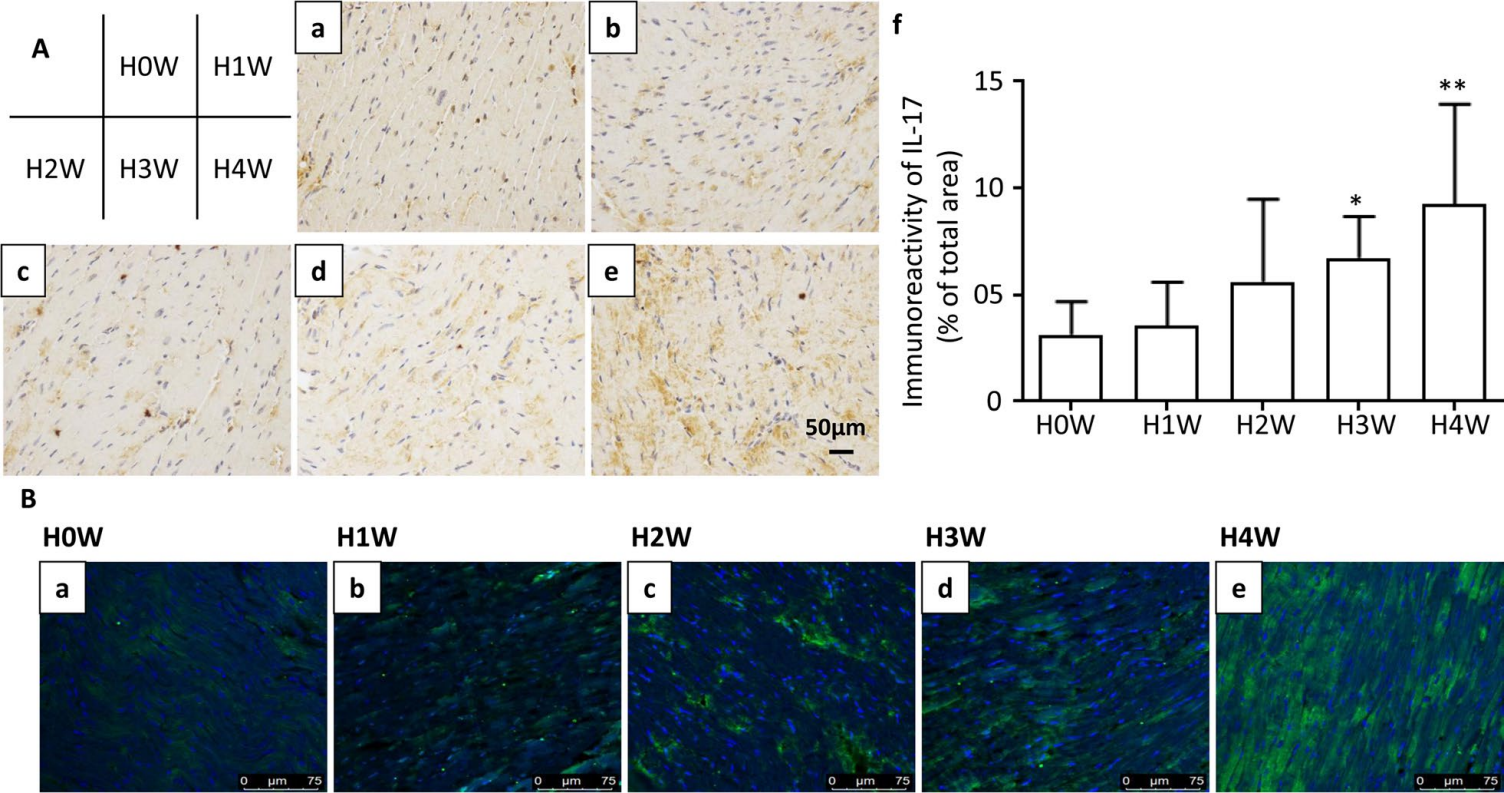
The expression of IL-17 is increased in the right ventricular tissue of mice during the hypoxia exposure. **A** Immunoreactivity for IL-17 in right ventricular tissue. (a, b, c, d, e) Immunohistochemical staining (brown) of IL-17 in right ventricular tissue sections. Scale bars: 50 μm. (f) Bar chart for IL-17 staining of right ventricular tissue sections from mice exposure to hypoxia for 0 week (H0W), 1 weeks (H1W), 2 weeks (H2W), 3 weeks (H3W), and 4 weeks (H4W). n = 8, *p = 0.0260, **p = 0.0077. **B** Immunofluorescent staining of IL-17 of representative histological sections from paraffin-embedded right ventricular tissues of mice. Blue for DAPI, green for IL-17. Scale bar = 75 μm

We have already known that RVHI level were gradually increased in a time-dependent manner after hypoxia exposure, and the expression of IL-17 in lung tissue and serum were also gradually increased [13]. To determine the effects of hypoxia exposure on the expression of IL-17 in the right ventricular tissue, RV tissues from mice exposed to hypoxia for 0, 1, 2, 3, and 4 weeks were collected. Immunohistochemical analysis (Fig. 2A) results revealed that the expression of IL-17 was gradually increased in a time-dependent manner after one week of hypoxia exposure, especially at the third and fourth weeks. The immunofluorescent analysis results (Fig. 2B) further confirmed that the expression of IL-17 was upregulated during the chronic hypoxia exposure.

### IL-17 aggravated hypoxia-induced right ventricular hypertrophy

**Fig. 3:**
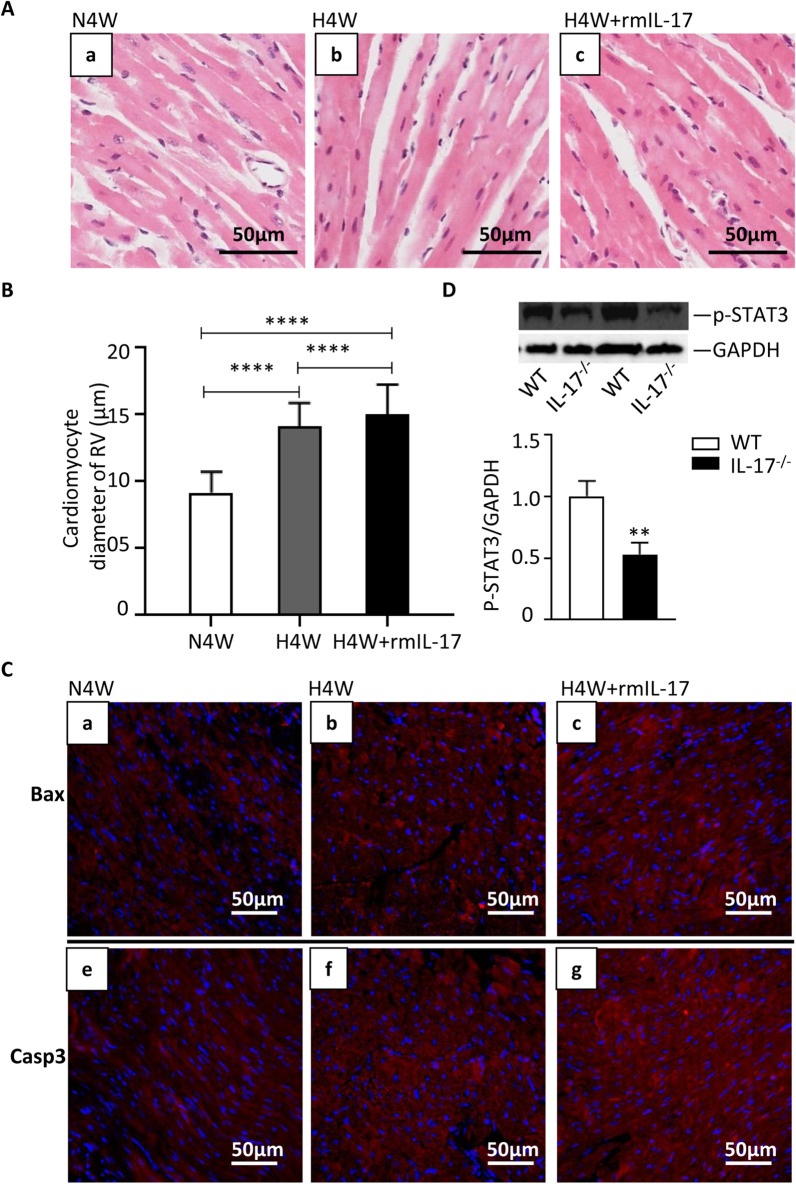
IL-17 aggravates hypoxia-induced right ventricular hypertrophy through activating STAT3. **A** Representative H&E staining of the right ventricular tissue sections derived from mice under normoxia (N4W, n = 7) or hypoxia (H4W, n = 8) for 4 weeks with rmIL-17 (H4W + rmIL-17, n = 6) or not. Scale bars: 50 μm. **B** Bar chart for cardiomyocytes diameter in the right ventricle of each group. ****p < 0.0001. **C** Immunofluorescent staining of Bax and caspase-3 of representative histological sections from paraffin-embedded mice right ventricular tissues of each group. Blue for DAPI, red for Bax and caspase-3. Scale bar = 50 μm. **D** Western blot analysis of p-STAT3 in right ventricular tissue of wide type mice and IL-17 knockout mice (n = 6) and the statistical bar graph. **p = 0.0063

From the above results, we established that IL-17 could induce right ventricular hypertrophy under normoxia and IL-17 in RV tissue was upregulated during the chronic hypoxia exposure. Our previous data showed that IL-17 aggravated hypoxia-induced right ventricular hypertrophy with elevated RVHI and RV/body weight ratio [13]. Cardiomyocyte hypertrophy and cardiomyocyte apoptosis are two different cellular events in ventricular hypertrophy. Then, we investigated the effect of IL-17 on the cardiomyocyte diameter and apoptosis under hypoxia. Figure 3A illustrates the results of the representative H&E staining of mice under normoxia, hypoxia treated with IL-17 or not for four weeks (N4W, H4W, H4W + rmIL-17). The cardiomyocyte diameter was measured as an indicator of ventricular hypertrophy (Fig. 3B). The hypoxia-exposed mice had a significantly larger cardiomyocyte diameter than the normoxia mice, which was further promoted by the treatment with rmIL-17. The cardiomyocyte apoptosis was investigated by detecting apoptosis-related gene Bax and caspase-3, as showed in Fig. 3C, the hypoxia-exposed mice had a higher expression of Bax and caspase-3, which was further upregulated by the treatment with rmIL-17. These data show that rmIL-17 significantly aggravated the hypoixa-triggered hypertrophy and apoptosis of individual cardiomyocytes in RV.

### The role of STAT3 in the IL-17 induced-right ventricular hypertrophy

Then, we aimed to better understand the potential mechanism of IL-17 induced-right ventricular injury. We have proved previously that IL-17 deficiency prevented development of hypoxia-induced pulmonary hypertension and right ventricular hypertrophy [13], results in the present study showed that the expression of p-STAT-3 in RV tissues from the IL-17-deficiency mice was lower than that in the wild-type mice (Fig. 3D), indicating that IL-17 may induce right ventricular hypertrophy through STAT-3 activation.

### The role of IL-17/STAT-3 in the cardiomyocyte injury

**Fig. 4:**
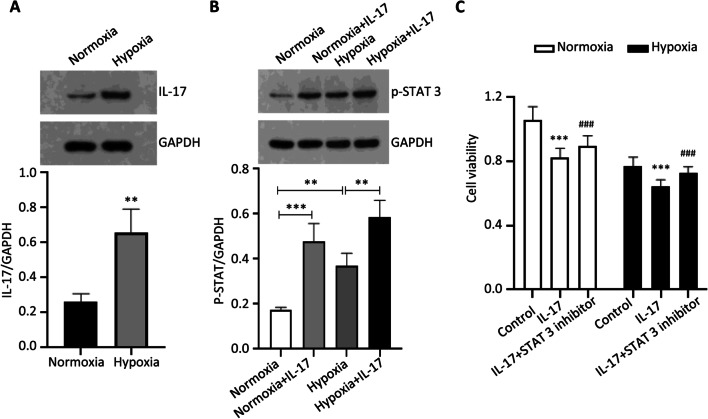
IL-17 inhibits cardiomyocyte viability via STAT3 under both normoxia and hypoxia condition. **A** Western blot analysis of IL-17 expression in cardiomyocytes after hypoxia exposure. **p < 0.01. **B** The protein expression of p-STAT3 in cardiomyocytes when treated with hypoxia exposure or/and IL-17. **p < 0.01, ***p < 0.001. **C** Cardiomyocyte viability measured by CCK-8 after treated with IL-17 or STAT3 inhibitor under normoxia and hypoxia. ***p < 0.001 (vs control group), ^###^*p* < 0.001 (vs IL-17 group)

Cardiomyocyte injury plays an important role during the right ventricular hypertrophy, and hypoxia could cause cardiomyocyte injury [18], which is in agreement with our results. In this study, the expression of IL-17 (Fig. 4A) and p-STAT-3 (Fig. 4B) after hypoxia exposure was upregulated. Notably, the expression of p-STAT-3 was further upregulated by the IL-17 treatment (Fig. 4B). As can be observed in Fig. 4C, cardiomyocyte viability was inhibited by IL-17 under both normoxic and hypoxic conditions, which was reversed by the STAT-3 inhibitor.

**Fig. 5:**
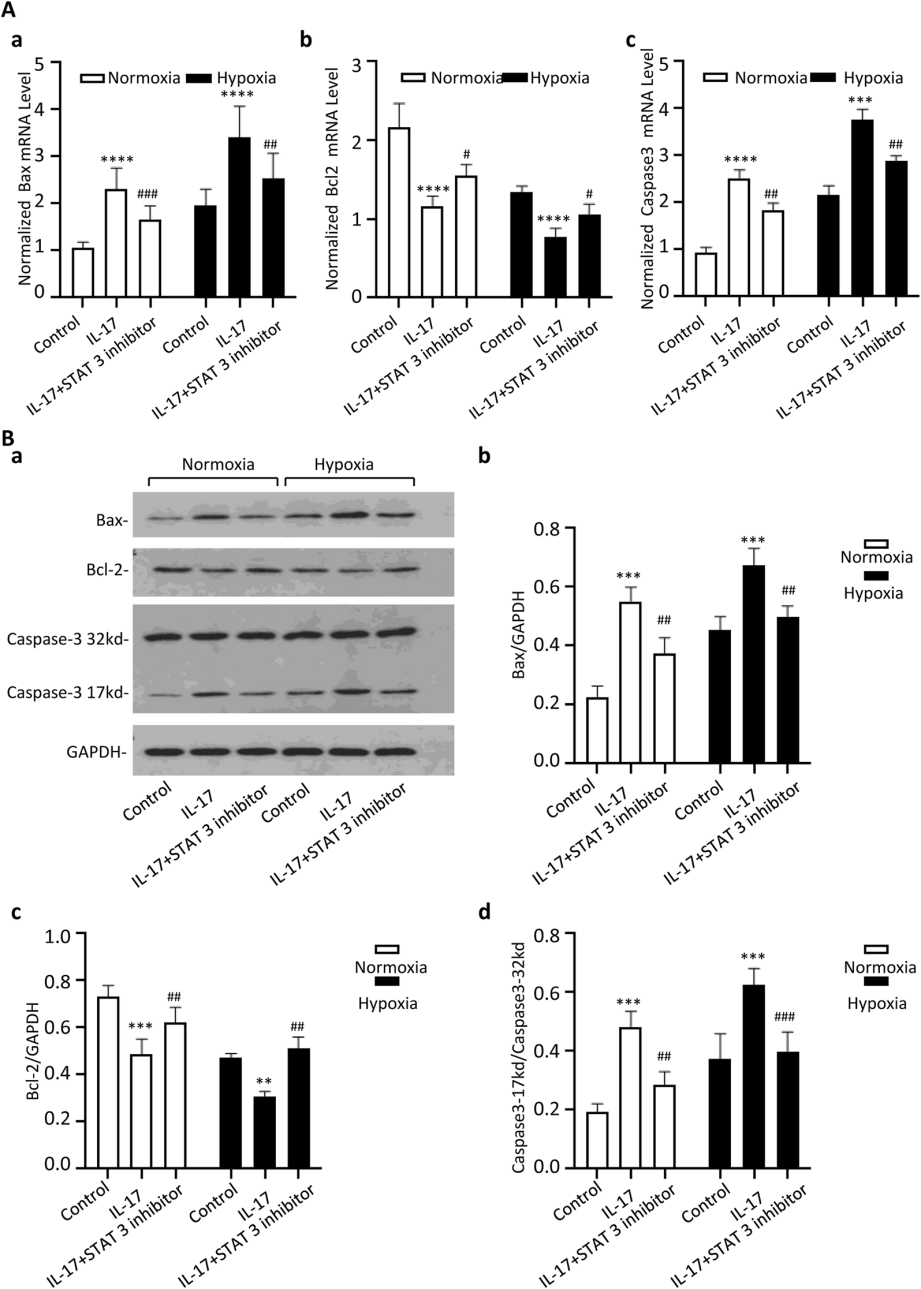
IL-17 induces cardiomyocyte apoptosis via STAT3 under both normoxia and hypoxia condition. (**A**, a, b, c) the normalized mRNA level of Bax, Bcl-2 and caspase3 after treated with IL-17 or STAT3 inhibitor under normoxia and hypoxia, respectively. ***p < 0.001, ****p < 0.0001 (vs control group). ^#^*p* < 0.05, ^##^*p* < 0.01, ^###^*p* < 0.001 (vs IL-17 group). **B** the protein expression of Bax, Bcl-2 and caspase3 after treated with IL-17 or STAT3 inhibitor under normoxia and hypoxia. (a) Western blot analysis of Bax, Bcl-2 and caspase-3. (b, c, d) Statistical bar graph for expression of Bax, Bcl-2 and caspase-3 respectively. **p < 0.01, ***p < 0.001 (vs control group). ^##^*p* 0.01, ^###^*p* < 0.001 (vs IL-17 group)

The effect of IL-17 on cardiomyocyte apoptosis was also investigated by detecting apoptosis-related genes. Bax and Bcl-2 belong to the Bcl-2 gene family, Bax is a pro-apoptotic regulator, whereas Bcl-2 is an anti-apoptotic regulator, and caspase-3 is a critical executioner of apoptosis. As illustrated in Fig. 5, IL-17 upregulated the expression of Bax and caspase-3 but downregulated the expression of Bcl-2 under normoxic and hypoxic conditions, which was reversed by the STAT-3 inhibitor. These data indicate that IL-17 inhibited cardiomyocyte viability and induced cardiomyocyte apoptosis via STAT-3 under both normoxic and hypoxic conditions.

## Discussion

In this study, we obtained the following main findings: (1) IL-17 induced RVH even under normoxia; (2) The expression of IL-17 in RV tissues during the chronic hypoxia exposure was upregulated in a time-dependent manner; (3) IL-17 further aggravated hypoxia-induced right ventricular hypertrophy; (4) IL-17 might participate in RVH through STAT3 activation; and (5) IL-17 induced cardiomyocyte injury and further aggravated hypoxia-induced cardiomyocyte injury by activating STAT3. To our knowledge, this is the first report to demonstrate that IL-17 is involved in RVH, suggesting a therapeutic potential target and diagnostic biomarker of IL-17 for RVH.

Several previous studies showed that IL-17 plays an important role in ventricular remodeling, but almost all of them focused on the left ventricle. In heart failure models, the levels of circulating CD4^+^ T cells and CD4^+^ T cells infiltration in the LV were significantly increased, and the expression of IL-17 was upregulated. Levels of fibrosis and collagen deposition were increased after IL-17 treatment [9]. In post- myocardial infarction mouse remodeling, IL-17 significantly aggravated both early- and late-phase left ventricular remodeling, but IL-17 deficiency had the opposite effect [11]. IL-17 did not participate in the early post-MI inflammatory process, but it had a specific role in the late remodeling stages by enhancing neutrophil and macrophage infiltration [19]. Additionally, the blockade of the IL-17 pathway was reported to alleviate late post-AMI remodeling [20]. In a myocarditis mouse model, IL-17 was essential for the development of the heart muscle enlargement, the heart ventricles dilatation, and the remodeling of LV during the process of transition from myocarditis to dilated cardiomyopathy [21, 22]. An in vitro study, showed that IL-17 directly induced apoptosis of cardiomyocytes [23]. In another investigation, IL-17 induced chemokine production by cardiac fibroblasts, resulting in neutrophil and macrophage infiltration in the heart, which critically involved in the pathogenesis of inflammatory dilated cardiomyopathy [24]. In cultured cardiac fibroblasts, IL-17 promoted the proliferation of fibroblasts, and upregulated the expression of ADAMTS-1, MMP-2, and collagen subtypes I and III, leading to fibrosis and collagen deposition [25].

So far, very few studies have investigated the role of IL-17 in right heart diseases. In one of them, IL-17 was upregulated in arrhythmogenic right ventricular cardiomyopathy which was characterized by fibrofatty remodeling [26]; however, but it did not examine its role in right ventricular cardiomyopathy and the possible mechanisms. These previous results are consistent with our findings, we confirmed that the expression of IL-17 in the RV tissue during the chronic hypoxia exposure was upregulated in a time-dependent manner. Furthermore, we also found that IL-17 induced RVH under normoxia and aggravated hypoxia-induced right ventricular hypertrophy.

Cardiomyocyte hypertrophy and cardiomyocyte apoptosis are two different cellular events in ventricular hypertrophy, and many factors can cause both cardiomyocyte hypertrophy and apoptosis, and hypertrophic cardiomyocyte can turned into apoptotic cell [27, 28]. Cardiomyocyte apoptosis is a well-known key cellular event during ventricular hypertrophy [2]. While cardiomyocytes apoptosis is rare in the normal heart, apoptotic rates increase significantly in human heart failure [29, 30]. Apoptosis rates in animal models were established to vary widely, but the potential of hypoxia to induce cardiomyocyte apoptosis is known [31]. Cardiomyocyte loss and subsequent reparative fibrotic healing lead to myocardial dysfunction and remodeling [32]. Nevertheless, previous studies showed that cardiomyocyte apoptosis can be manipulated and even reversed [33, 34]. Therefore, it is important to elucidate the mechanisms of cardiomyocyte apoptosis, which have not yet been fully clarified. In the present investigation both IL-17 and hypoxia caused cardiomyocyte apoptosis. Apoptosis-related proteins, such as Bcl-2, Bax and caspase-3, are known to be critically involved in apoptosis. Our present results indicated that IL-17 markedly downregulated Bcl-2, upregulated Bax and caspase-3, and reduced the survival rate of cardiomyocytes. Meanwhile, the treatment with STAT-3 inhibitor reversed the aforementioned effects. These indicated that IL-17 may exert its pro-apoptotic effects through STAT-3 expression regulation.

IL-17 (usually known as IL-17A), is an important proinflammatory cytokine mainly produced by Th17 cells that binds to a heteromeric receptor, composed of IL-17RA and IL-17RC subunits, which activates the canonical NF-κB, MEK-ERK1/2, PI3K-Akt, JNK, and p38 MAPK pathways [35]. The data obtained in this study showed that the expression of p-STAT3 in IL-17-deficient mice RV tissue was downregulated, indicating that IL-17 may induce right ventricular hypertrophy through activating STAT3. Further, our present results revealed that the applied STAT-3 inhibitor reversed the pro-apoptotic effect of IL-17; hence, IL-17/STAT-3 pathway plays an important role in the cardiomyocyte apoptosis.

There are several limitations in this study. First, here, we did not compare the difference between RV and LV. Second, RV hypertrophy biomarkers, myocardial inflammation, myocardial fibrosis, right ventricular structure and function features was not assessed in this study. Third, cardiomyocyte-specific IL-17 deficiency mice, other pulmonary hypertension animal models and right ventricular failure model were not used to investigate the role of IL-17 in RVH. Last, the deep pathogenesis mechanisms need to be further investigated. Certainly, all of these are our future research directions. In our future investigations, we will isolate and culture RV and LV primary cardiomyocytes to establish the pathogenetic mechanisms of IL-17 in RVH using different animal models.

## Conclusions

In conclusion, this work provides preliminary evidence that IL-17 is a mediator in the pathogenesis RVH, which might be considered as a potential novel anti-inflammation therapeutic strategy or diagnostic biomarker for RVH.

## Supplementary Information

**Additional file 1**. Full-length blots of WB results and flowcharts of in vivo experiments.

## Data Availability

The data used to support the findings of this study are available from the corresponding author upon request.
