# Supplementary material for: Interleukin-17 aggravates right ventricular remodeling via activating STAT3 under both normoxia and hypoxia
Source: BMC Cardiovasc Disord. 2021 May 21;21:249. doi: 10.1186/s12872-021-02069-4 (PMC8139008; doi:10.1186/s12872-021-02069-4)

Interleukin-17 aggravates right ventricular remodeling via activating STAT3 under both normoxia and hypoxia

Huang Jing[1#], Wei Zhang[2#], Cai-lian Zhang[3], Lei Wang[4 *]

1. Department of Rheumatism and Immunology, First Affiliated Hospital of Xi'an Jiaotong University, Xi'an 710061, P.R. China

2. Department of Emergency Medicine, First Affiliated Hospital of Xi'an Jiaotong University, Xi'an 710061, P.R. China

3. Department of Pulmonary and Critical Care Medicine, Yanan University Affiliated Hospital, Yanan 716000, P.R. China

4. Department of Pulmonary and Critical Care Medicine, Second Affiliated Hospital of Xi'an Jiaotong University, Xi'an 710004, P.R. China

* Corresponding author

Address for reprint requests and other correspondence:

Dr. Lei Wang

No. 157 Xiwu Road, Xin Cheng District, Xi'an 710004, P.R. China

Tel: 029-87679973

Email: wl860806wb@163.com

Running title: IL-17 and right ventricular remodeling

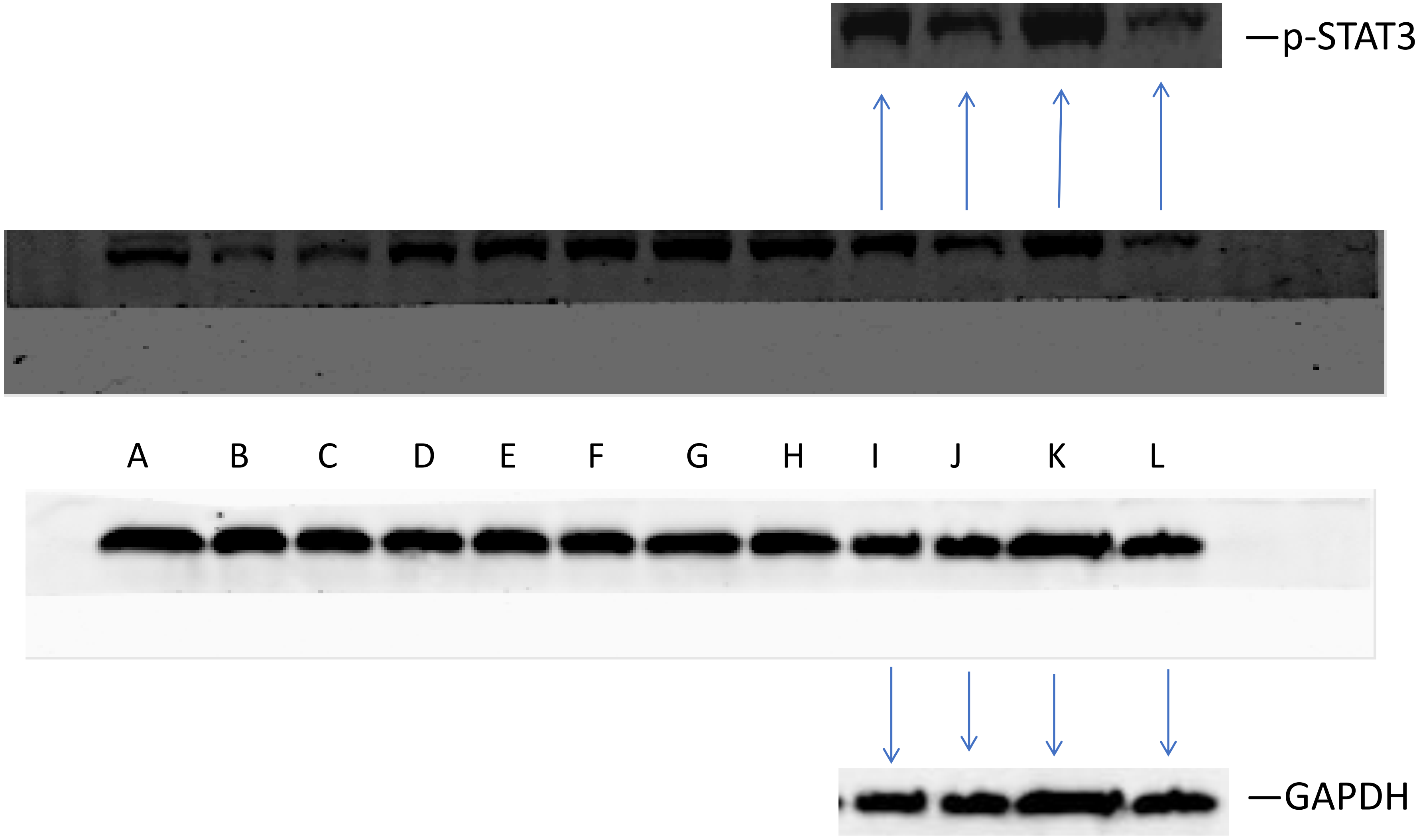

Figure 3C

A: WT 1
B: IL17 KO 1
C: IL17 KO 2
D: WT 2
E: WT 3
F: IL17 KO 3
G: WT 4
H: IL17 KO 4
I: WT 5
J: IL17 KO 5
K: WT 6
L: IL17 KO 6

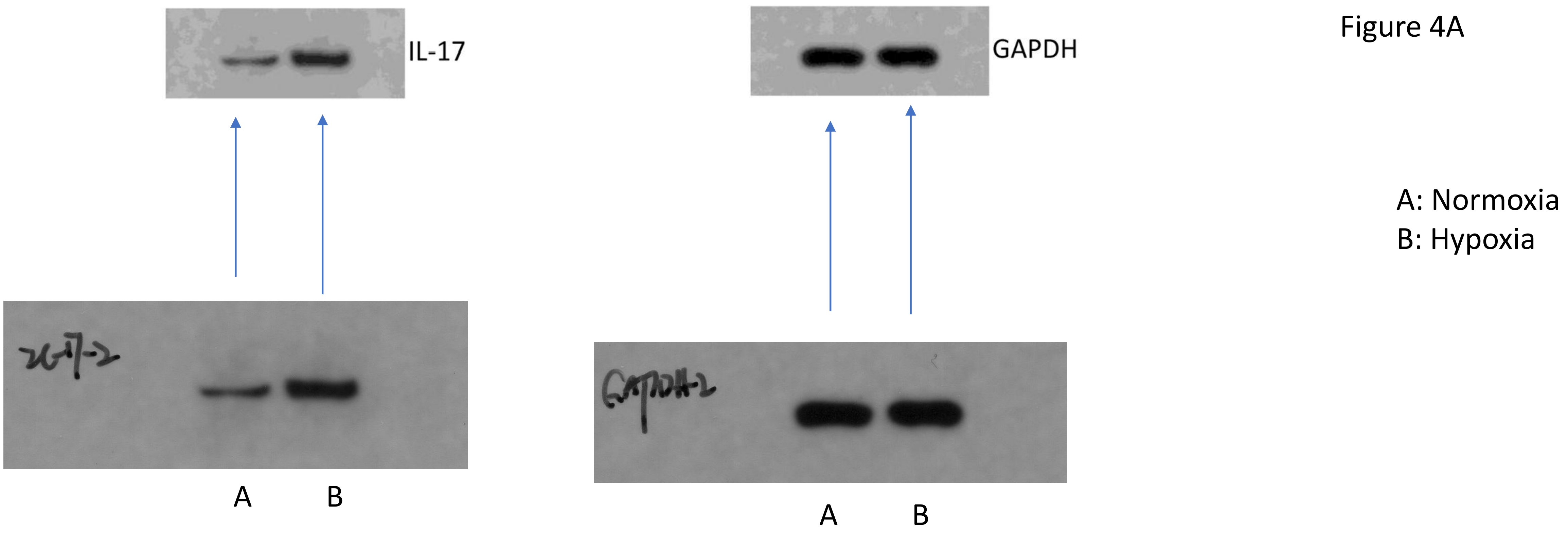

Figure S1 IL-17 expression in cardiomyocytes after hypoxia exposure.

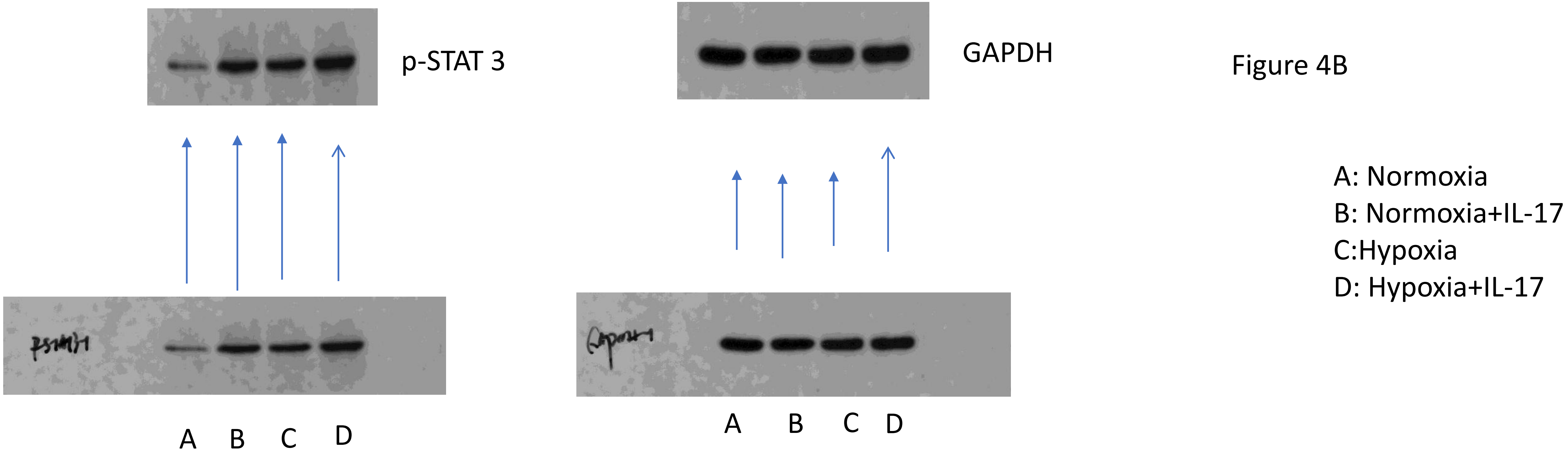

Figure S2 Expression of p-STAT3 in cardiomyocytes when treated with hypoxia exposure or/and IL-17.

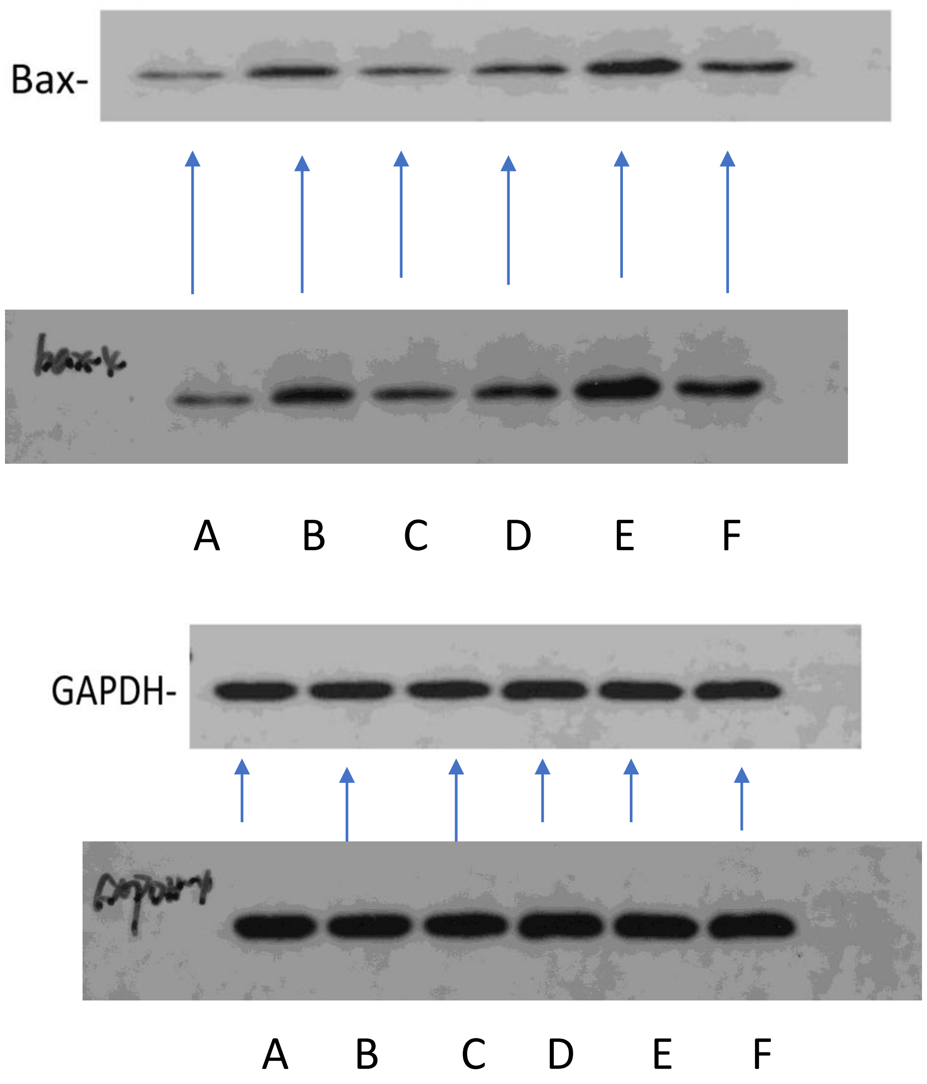

F5Ba

Normoxia
A: Control
B: IL-17
C: IL-17+STAT 3 inhibitor
Hypoxia
D: Control
E: IL-17
F: IL-17+STAT 3 inhibitor

Figure S3 Expression of Bax after treated with IL-17 or STAT3 inhibitor under normoxia and hypoxia.

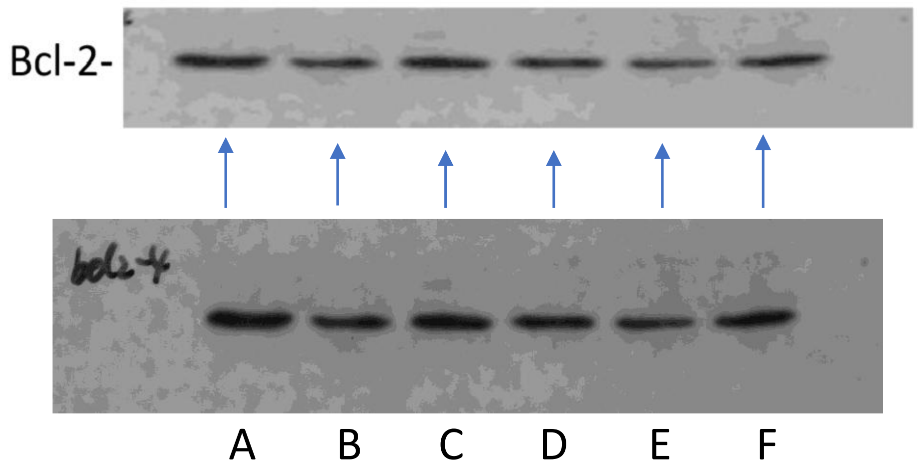

F5Ba

Normoxia
A: Control
B: IL-17
C: IL-17+STAT 3 inhibitor
Hypoxia
D: Control
E: IL-17
F: IL-17+STAT 3 inhibitor

Figure S4 Expression of Bcl-2 after treated with IL-17 or STAT3 inhibitor under normoxia and hypoxia.

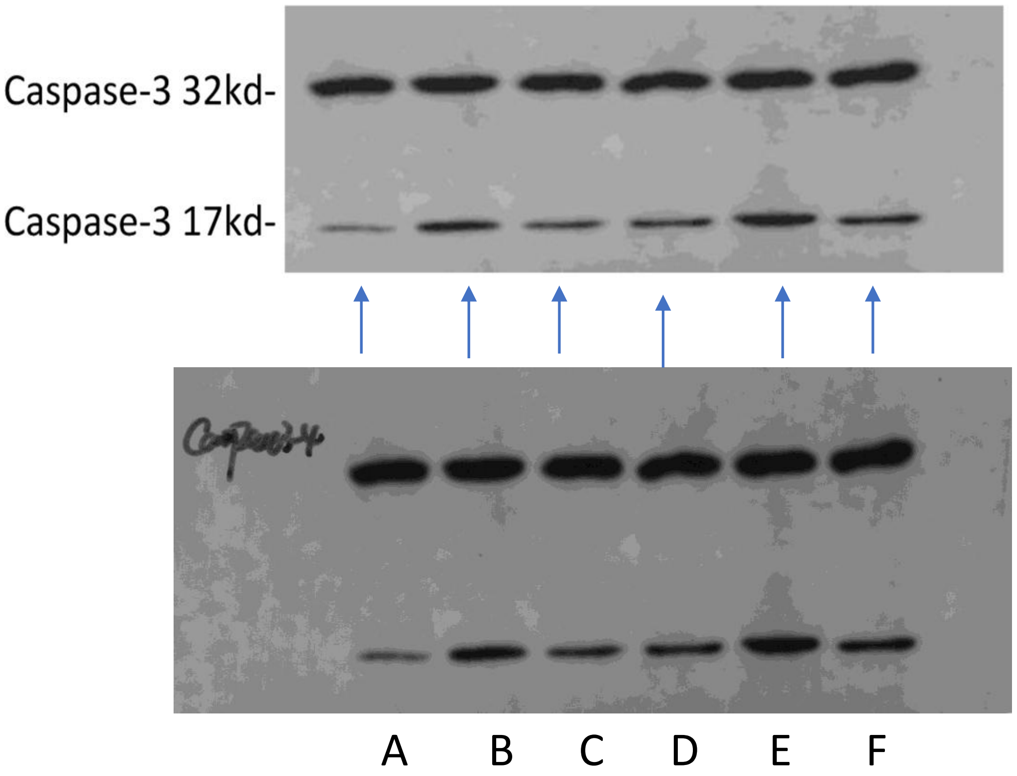

F5Ba

Normoxia
A: Control
B: IL-17
C: IL-17+STAT 3 inhibitor
Hypoxia
D: Control
E: IL-17
F: IL-17+STAT 3 inhibitor

Figure S5 Expression of Caspase3 after treated with IL-17 or STAT3 inhibitor under normoxia and hypoxia.

Flowchart of in vivo experiments

Experiment of Figure 1

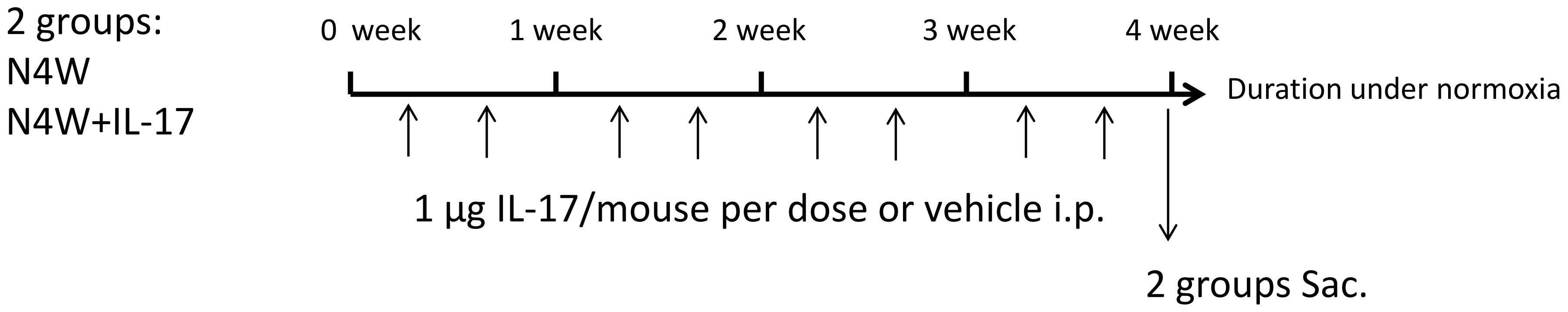

Experiment of Figure 2

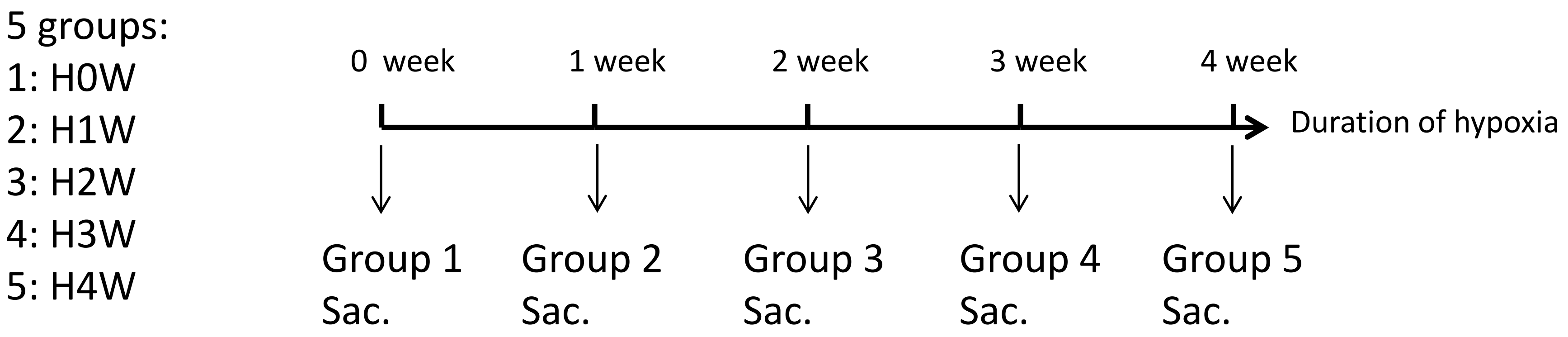

Flowchart of in vivo experiments

Experiment of Figure 3

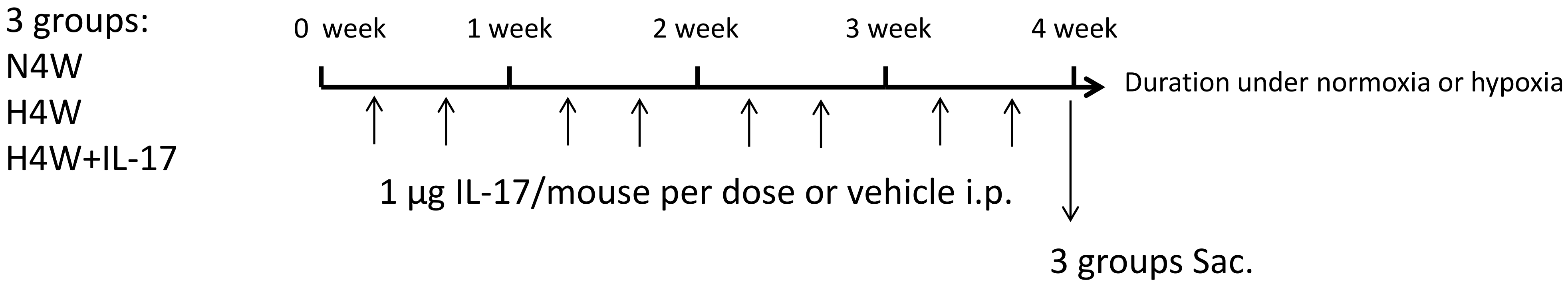

Supplement: Supplementary file 1 — Additional file 1. Full-length blots of WB results and flowcharts of in vivo experiments. [file 12872_2021_2069_MOESM1_ESM.pdf]
